# Uniformly dispersed platinum-cobalt alloy nanoparticles with stable compositions on carbon substrates for methanol oxidation reaction

**DOI:** 10.1038/s41598-017-10223-2

**Published:** 2017-09-12

**Authors:** Hui Liu, Chengyin Li, Dong Chen, Penglei Cui, Feng Ye, Jun Yang

**Affiliations:** 1 State Key Laboratory of Multiphase Complex Systems, Institute of Process Engineering, Chinese Academy of Sciences, Beijing, 100190 China; 2 Center for Mesoscience, Institute of Process Engineering, Chinese Academy of Sciences, Beijing, 100190 China; 3 University of Chinese Academy of Sciences, No. 19A Yuquan Road, Beijing, 100049 China

## Abstract

Alloying platinum (Pt) with suitable transition metals is effective way to enhance their catalytic performance for methanol oxidation reaction, and reduce their cost at mean time. Herein, we report our investigation on the synthesis of bimetallic platinum-cobalt (PtCo) alloy nanoparticles, their activation, as well as the catalytic evaluation for methanol oxidation reaction. The strategy starts with the synthesis of PtCo alloy nanoparticles in an organic medium, followed by loading on carbon substrates. We then remove the capping agent by refluxing the carbon-supported PtCo particles in acetic acid before electrochemical measurements. We emphasize the change in composition of the alloys during refluxing process, and the initial PtCo alloys with Pt/Co ratio of 1/2 turns into stable alloys with Pt/Co ratio of 3/1. The final Pt_3_Co particles have uniform distribution on carbon substrates, and exhibit activity with 2.4 and 1.5 times of that for commercial Pt/C and PtRu/C for methanol oxidation reaction.

## Introduction

One of the major problems with direct methanol fuel cells (DMFCs), which are considered to be most promising as power sources for portable devices, is the slow oxidation kinetics of methanol oxidation reaction (MOR) at the anode^1–5^. In addition, the MOR involves a complex network of reactions with many possible side products (6-electrons transferred in methanol oxidation *versus* only 2-electrons are transferred in hydrogen oxidation) especially at low operating temperatures^6–8^. Therefore, electrocatalysts with higher activity for MOR are required in the case of DMFCs^9, 10^. Platinum (Pt) is the standard catalyst used for the oxidation of small organic molecules including methanol. There have been many studies on MOR catalyzed by the presence of Pt, including the determination of the optimal Pt particle size^11, 12^, shape^4, 13^, structure^14–17^, the effects of catalyst support^18^, and the development of alternative carbon supports to substitute for the commonly used acetylene black carbon, Vulcan XC-72^19^. Unfortunately, the situation is made worse by the rapid deactivation of the Pt catalyst surface by CO-like intermediates, which are formed during the stepwise dehydrogenation of methanol^20^.

The poisoning of Pt catalysts by CO-like intermediates is usually addressed by integrating Pt with one or more oxophilic metals. The improvement in these binary & ternary catalyst systems has been attributed to the collaborative action between the metals known as bi-functional catalysis^21, 22^: Methanol dehydrogenation occurs preferentially on the Pt sites and water dissociation occurs preferentially on the oxophilic metal sites. The adsorbed CO intermediates on the Pt sites can then be oxidized by the oxygen-containing species on the neighboring oxophilic metal sites. Although ruthenium (Ru) is often selected as the oxophilic metal in binary or multi-metallic (ternary & quaternary) catalyst formulations^23–30^, Ru, as a precious metal with an even rarer presence than Pt, can hardly reduce the cost of catalyst production^31, 32^. As a consequence, an alternative approach, which is based on the substitution of Ru by cobalt (Co) to form PtCo alloy catalysts, has been developed to resolve the problem associated with Ru metal. This strategy is validated by the fact that, upon alloying, the Co is known to change the position of the d-band center of Pt by modifying the electronic structure of neighboring Pt atoms^33^, thus affecting the bond strength of Pt-CO and promoting the cleavage of C-H bond at lower potential. Further, the possible presence of Co oxides at the surface of the catalysts may provide a source of oxygen, favorable for the oxidation of CO-like intermediates. In particular, in 2010, Rojas and co-workers systemically investigated the effect of Co in the efficiency of the MOR on carbon supported Pt by means of differential electrochemical mass spectrometry (DEMS)^34^. They found that alloying Pt with Co would significantly increase the CO_2_ efficiency, especially at lower potentials, region of great interest for the MOR. Over the past 15 years, a large number of PtCo alloy nanoparticles with different morphologies, e.g. spheres, wires, or ribbons have been produced by conventional wet-chemistry methods for catalyzing the electrooxidation of methanol^35–46^.

For the electrocatalysts prepared by wet-chemistry methods, the surfactants containing amino or thiol group^47, 48^, which are usually used to enhance the nanoparticle stability in solution, would result in poor electrocatalytic performance by occupying a large number of surface atoms. Therefore, before performing the electrochemical measurements, the electrocatalysts are usually activated by refluxing in acetic acid for period of time to remove the surface capping agents^49^. In this process, the chemical composition of the electrocatalysts, particularly for those containing active transition metals may be changed greatly, but rarely noticed. In this study, we report our investigation on the synthesis of bimetallic PtCo alloy nanoparticles, their activation, as well as the catalytic performance for MOR. Our study start with the synthesis of PtCo alloys with Pt/Co molar ratio of 1/2, followed by uniformly dispersion on carbon substrates. As we will demonstrate, the activation by acetic refluxing could significantly change the composition of PtCo alloys. After refluxing, the Pt/Co molar ratio in PtCo allys changes into 3/1 from 1/2 and keeps stable. The final Pt_3_Co alloy products uniformly disperse on carbon substrates display superior activity and stability for MOR, and their activity is much higher that of the commercial Pt/C and PtRu/C electrocatalysts from Johnson Matthey.

## Results and Discussion

**Figure 1:**
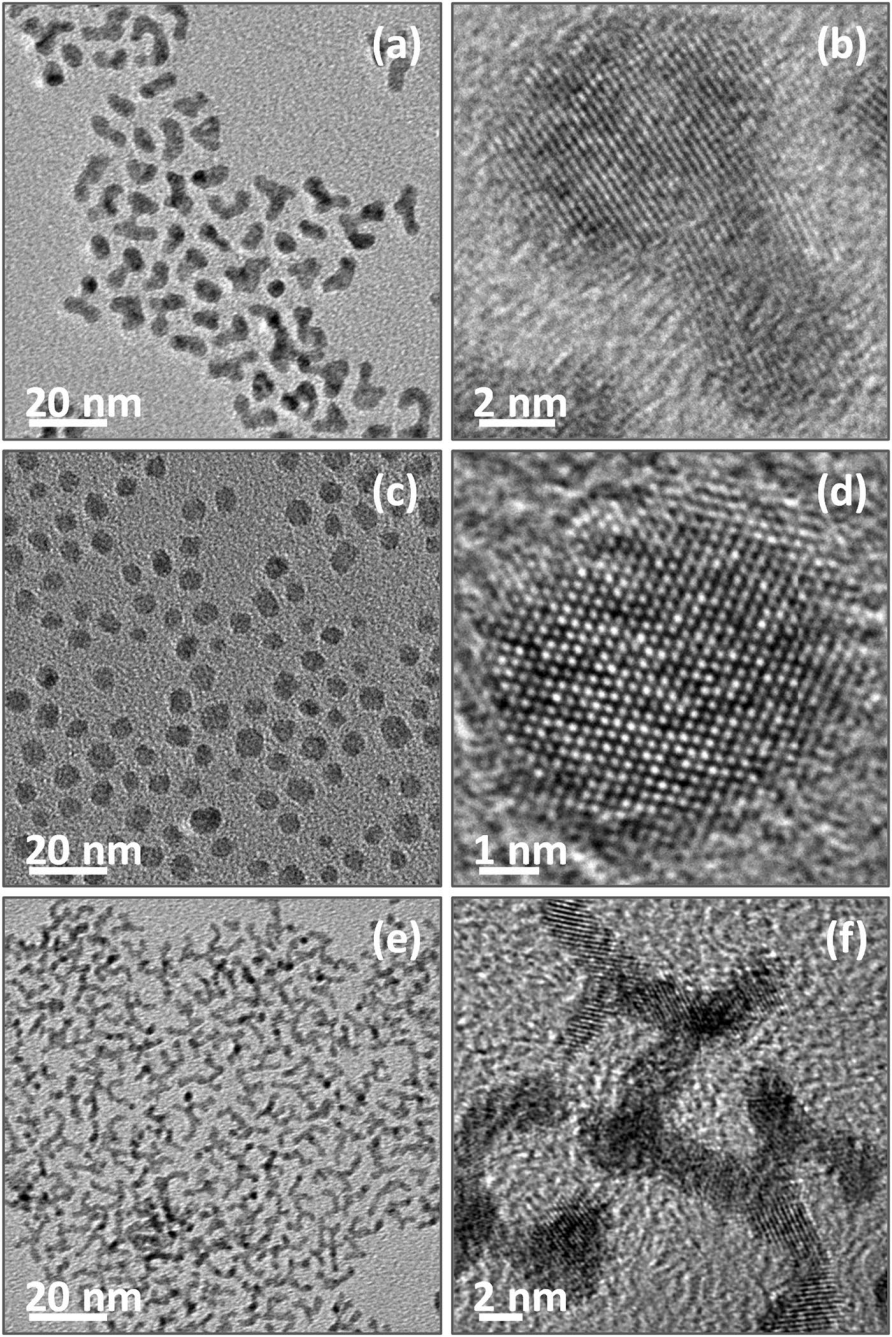
TEM images (**a**,**c**,**e**) and HRTEM images (**b**,**d**,**f**) of PtCo (**a**,**b**), Co (**c**,**d**), and Pt nanoparticles (**e**,**f**) as-prepared in 1-octadecene at elevated temperature.

At elevated temperature, the metal precursors could be efficiently reduced by oleylamine, which is the most commonly used reducing agent and stabilizing agent^50–54^, for the formation of metal nanoparticles. As displayed by Fig. 1a, the TEM image of the as-prepared bimetallic PtCo products shows that most of the particles have worm-like morphologies with ca. 3.1 nm in widths and 12.9 nm in lengths, which are remarkably different from spherical Co particles with average diameter of ca. 6.6 nm (Fig. 1c) or dendritic Pt particles with thin width of ca. 1.7 nm (Fig. 1e) obtained in same experimental conditions by reducing sole Co(acac)_2_ or Pt(acac)_2_ precursors, respectively. The differences in size and morphology are indirect evidences to indicate the formation of bimetallic alloy PtCo particles instead of a mixture of isolated Co and Pt nanoparticles. The high-resolution TEM (HRTEM) images (Fig. 1b,d and f) illustrate the lattice planes in these nanocrystals, confirming that bimetallic PtCo, monometallic Co and Pt are of high crystallinity.

**Figure 2:**
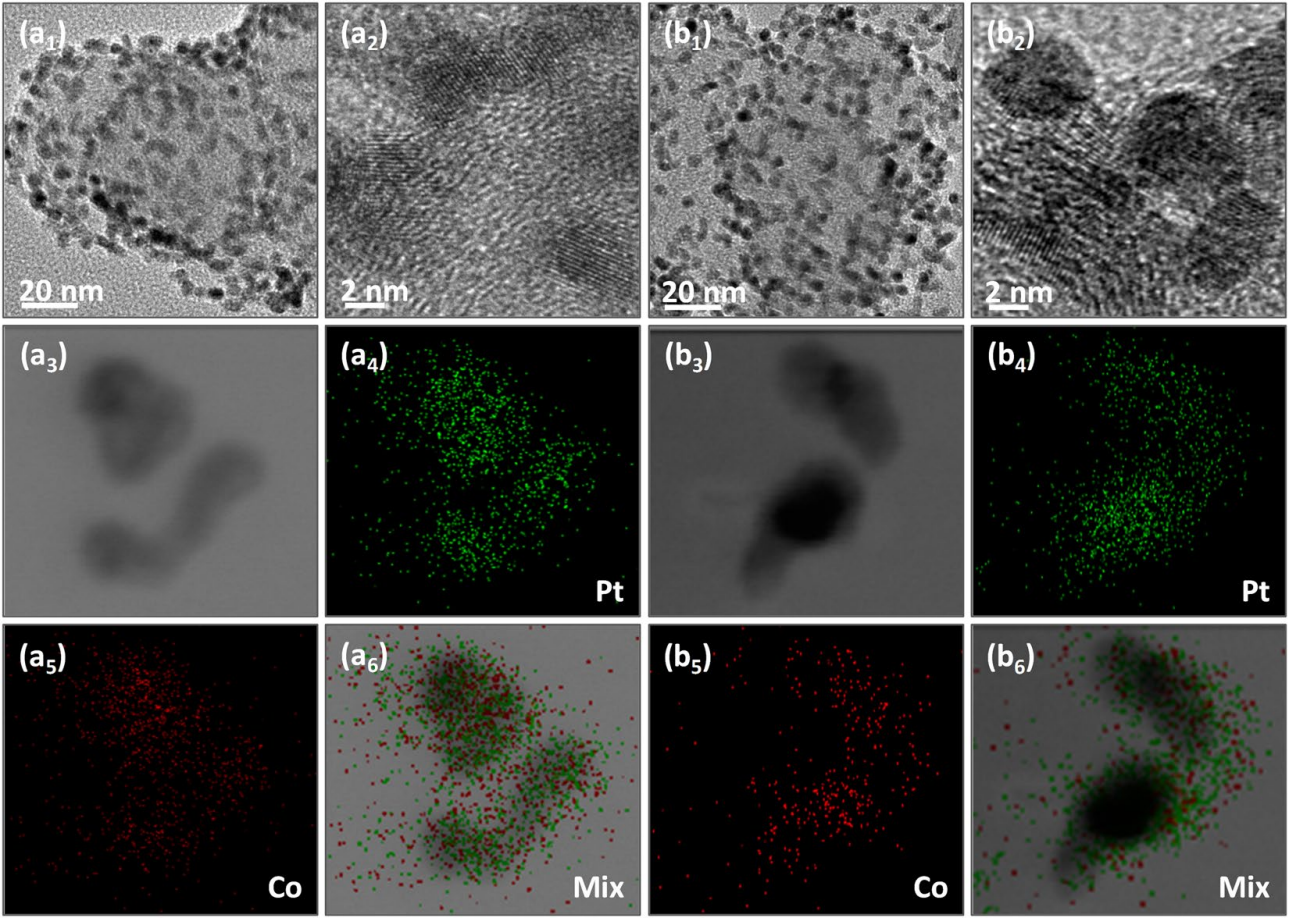
TEM images (**a**_**1**_,**b**_**1**_), HRTEM images (**a**_**2**_,**b**_**2**_), STEM images (**a**_**3**_,**b**_**3**_), elemental mappings (**a**_**4**_–**a**_**6**_,**b**_**4**_–**b**_**6**_) of carbon-supported PtCo nanoparticles before (**a**_**1**_–**a**_**6**_) and after (**b**_**1**_–**b**_**6**_) refluxing in acetic acid at 120 °C.

By conventional aging XC-72 carbon substrates (XC-72C) in PtCo colloidal solution in toluene, we can uniformly load the PtCo nanoparticles on the surface of carbon supports, as exhibited by Fig. 2a_1_ and a_2_ for the TEM and HRTEM images. The alloy nature of bimetallic PtCo nanoparticles could be confirmed by elemental mapping analyses (Fig. 2a_4_–a_6_) of two arbitrary single particles (Fig. 2a_3_), which reveal that the both Pt and Co signals are distributed uniformly throughout the entire particles. The XRD pattern shown in Figure S1a of Supplementary Information (SI) for the PtCo products supported on carbon supports indicates the existence of a homogeneously mixed crystal lattice, also suggesting the formation of bimetallic alloy nanoparticles. The EDX analysis (SI Figure S2a) produces a Pt/Co atomic ratio of 34/66, which is consistent with the molar ratio of the Pt and Co metal precursors. Before electrochemical measurements, we usually need to reflux the PtCo/C in acetic acid for a period of time to remove the capping agent from the particle surface^49^. The refluxing in acetic acid does not change the size and morphology of the PtCo nanoparticles (Fig. 2b_1_ and b_2_ for TEM and HRTEM images) as well as their alloy nature (Fig. 2b_3_–b_6_ for the elemental mapping analyses). The uniform distribution of the alloy PtCo nanoparticles on the surface of carbon substrates is also maintained, as evinced by Fig. 2b_1_ for the TEM image of large areas. However, after refluxing in acetic acid, the signal of Co in the elemental mappings turns into much weaker than that of the original PtCo/C (before refluxing), as manifested by the comparison between Fig. 2a_5_ and b_5_. The EDX analysis (SI Figure S2b) shows that the alloy PtCo particles have a Pt/Co atomic ratio of 77/23 after refluxing in acetic acid, demonstrating that the refluxing in acetic acid could result in significant loss of Co from the bimetallic PtCo alloy particles although their alloy nature could be retained. The XRD pattern (SI Figure S1b) shows that the alloy PtCo particles after refluxing in acetic acid have Pt_3_Co phase, which is in good agreement with the reference of face-centered cubic Pt_3_Co alloys (JCPDS Card No. 290499). The extension of refluxing time in acetic acid will not further alter the composition of alloy PtCo nanoparticles, indicating the PtCo alloy at molar ratio of 3/1 is stable in experimental conditions.

**Figure 3:**
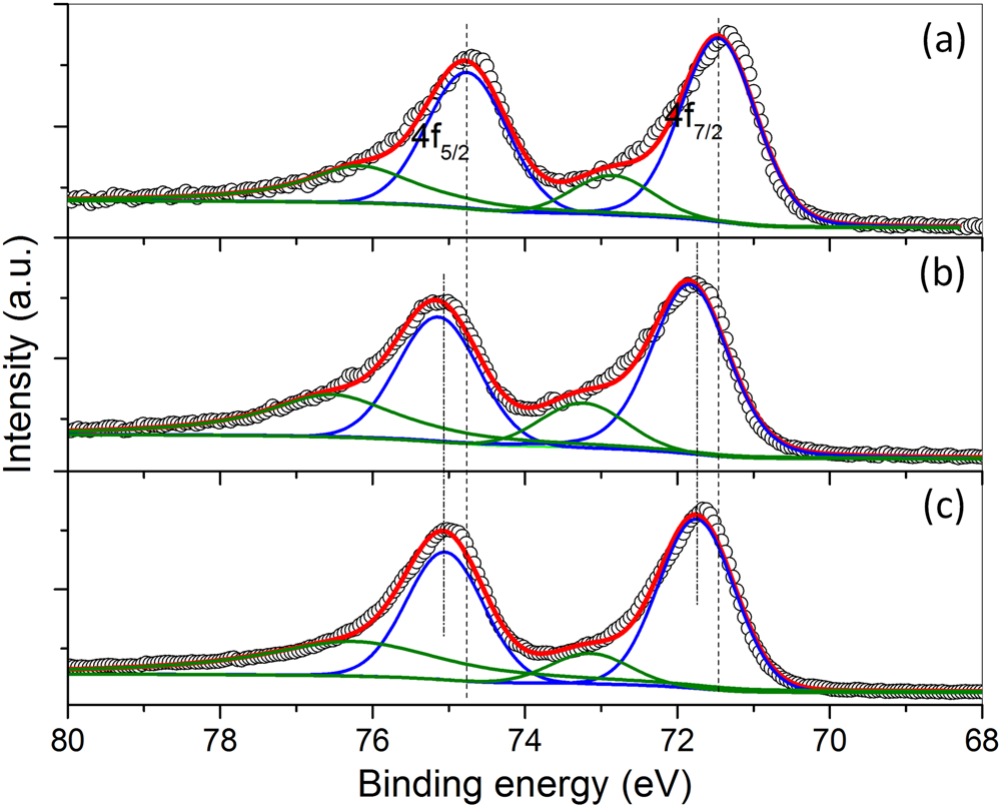
4 f XPS spectra of Pt in commercial Pt/C (**a**), PtCo alloys before (**b**), and after (**c**) refluxing in acetic acid at 120 °C for 3 h.

We examine the chemical state of Pt in PtCo alloy nanoparticles before and after refluxing in acetic acid using XPS and compare with that of commercial Pt/C catalysts. As shown in Fig. 3, the more intense doublet (at 71.5 and 74.8 eV for commercial Pt/C, 71.8 and 75.2 eV for PtCo/C before refluxing, and 71.7 and 75.1 eV for PtCo/C after refluxing, respectively) can be assigned to Pt at zero valent state, while the less intense double with binding energy of ca. 1.4 eV higher than the metallic Pt could be attributed to the Pt at oxidized state, e.g. PtO^44, 55, 56^. As observed by comparing the binding energies in Fig. 3a and b, we observe that the binding energies of Pt 4 f XPS peaks for PtCo/C before refluxing have a slight shift to higher values in comparison with those of commercial Pt/C catalysts. This shift might be induced by the 5d electron loss in Pt-based alloys, as demonstrated by Mukerjee and co-workers in their XANES study^57^, or by the work function changes, as reported in previous studies on Pt-Co and Pt-Ru nanoalloys^58^. After refluxing in acetic acid, the loss of Co in PtCo alloy nanoparticles results in tiny back-shift for the Pt 4 f binding energies (Fig. 3c). However, the 4 f binding energies in Pt_3_Co/C (PtCo/C after refluxing) is still slightly higher than those of commercial Pt/C catalysts due to the presence of Co in the alloys. Analogous to the bimetallic Cu-Pd system^59^, the electronic interaction between Co and Pt in Pt_3_Co alloy nanoparticles would lead to a lowered d-band center of Pt, which weakens the chemisorption of some small molecules, e.g. H_2_ and CO, on Pt surface, and thus facilitate the performance of the Pt catalysts for MOR.

**Figure 4:**
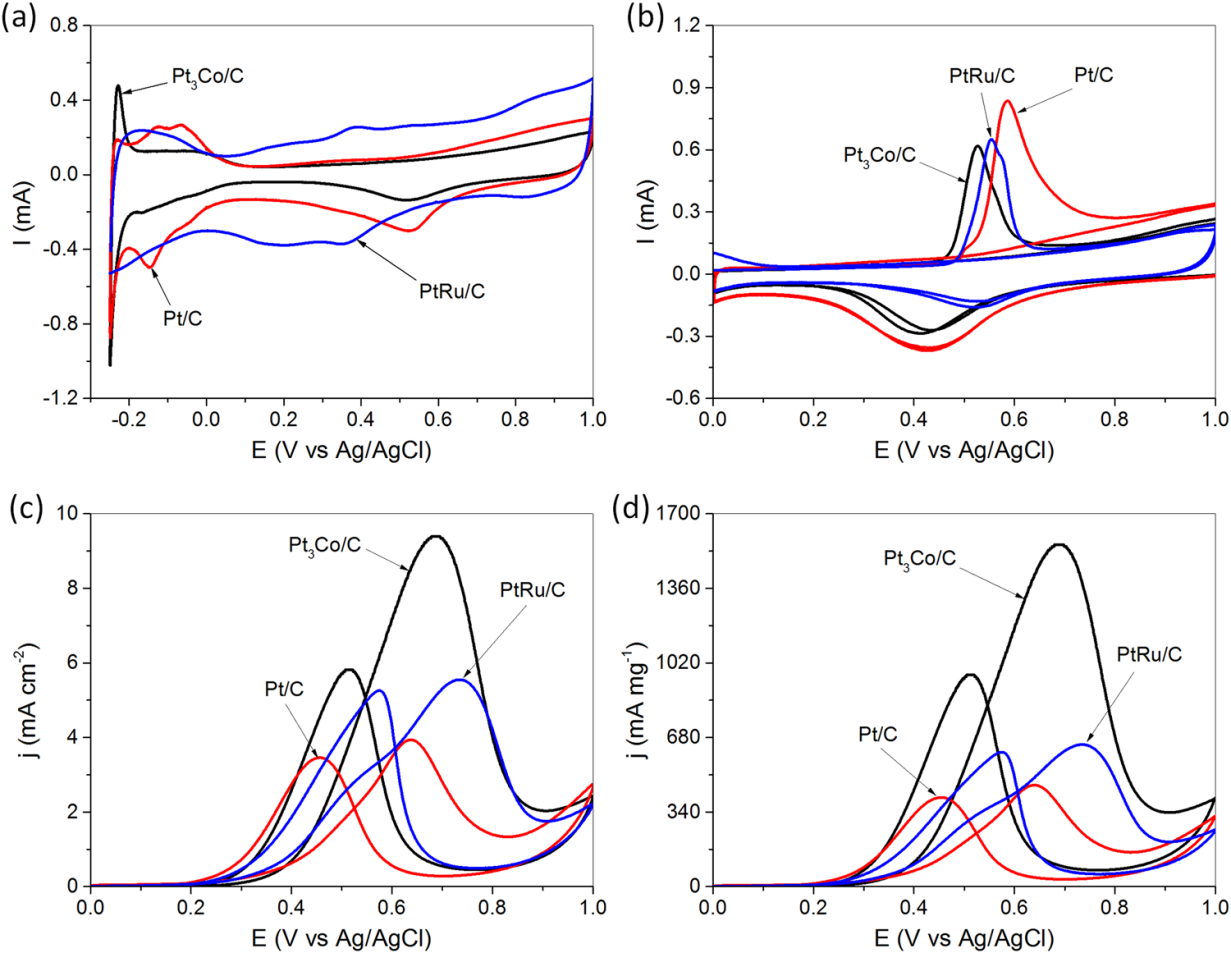
Cyclic voltammograms of Pt_3_Co/C, commercial Pt/C, and commercial PtRu/C catalysts in argon-purged HClO_4_ electrolyte (0.1 M) at a scan rate of 50 mV s^−1^ (**a**); room temperature CO stripping cyclic voltammograms of Pt_3_Co/C, commercial Pt/C, and commercial PtRu/C catalysts in argon-purged HClO_4_ electrolyte (0.1 M) at a scan rate of 20 mV s^−1^ (**b**); ECSA-based cyclic voltammograms of Pt_3_Co/C, commercial Pt/C, and commercial PtRu/C catalysts in argon-purged HClO_4_ (0.1 M) with methanol (1 M) at a scan rate of 20 mV s^−1^ (**c**); mass-based cyclic voltammograms of Pt_3_Co/C, commercial Pt/C, and commercial PtRu/C catalysts in argon-purged HClO_4_ (0.1 M) with methanol (1 M) at a scan rate of 20 mV s^−1^ (**d**).

Upon the above-mentioned analyses, we then carry out the electrochemical measurements to evaluate the catalytic performance of final Pt_3_Co/C products, which have stable alloy compositions. Fig. 4a shows the cyclic voltammograms of Pt_3_Co/C, commercial Pt/C, and commercial PtRu/C in 0.1 M HClO_4_ electrolyte, which is used to determine the electrochemically active surface areas (ECSAs) of the corresponding catalysts. The specific ECSAs (based on Pt mass) calculated by integrating the charge associated with the hydrogen adsorption/desorption potential region after double-layer correction, are 139.8 m^2^ g^−1^ for Pt_3_Co/C, 103.3 m^2^ g^−1^ for commercial Pt/C, and 67.2 m^2^ g^−1^ for commercial PtRu/C, respectively. Although the overall size of Pt_3_Co sample is bigger than that of commercial Pt/C (ca. 3.5 nm, SI Figure S3a) and PtRu/C catalysts (ca. 3 nm, SI Figure S3b), the higher ECSAs of Pt_3_Co alloy particles suggest that there are more active sites in Pt_3_Co alloys than those in commercial catalysts, most likely due to the presence of active edges/corner atoms in their worm-like morphologies with thin sizes in width.

Figure 4b shows the room temperature CO stripping cyclic voltammograms of Pt_3_Co/C, commercial Pt/C, and commercial PtRu/C catalysts in argon-purged HClO_4_ electrolyte. As observed, the peak potential of CO-stripping on Pt_3_Co/C alloy particles is 0.53 V, lower than that on commercial Pt/C (0.58 V) or PtRu/C catalysts (0.55 V) due to the down-shift of d-band center of Pt, as evinced by the XPS analyses in Fig. 3, which weakens the interaction between Pt and CO^59^. The lower stripping potential suggests that the Pt_3_Co alloy particles have a higher CO tolerance than the commercial Pt/C and PtRu/C catalysts.

Figure 4c shows the cyclic voltammograms of MOR on Pt_3_Co/C, commercial Pt/C, and commercial PtRu/C catalysts. The potential window is 0–1 V with a sweeping rate of 20 mV s^−1^, and the current densities as-measured are normalized by the ECSAs. As expected, the Pt_3_Co/C exhibits much higher activity for MOR than Pt/C and PtRu/C due to the electronic interaction between the Pt and Co components in the alloys. The peak current density in the forward scan, which are often used to evaluate the performance of catalysts for MOR^60, 61^, for Pt_3_Co/C is 9.44 mA cm^−2^, 2.4 and 1.5 times of that for Pt/C (3.97 mA cm^−2^) and PtRu/C (5.53 mA cm^−2^), respectively. In addition, the I_f_/I_b_ (ratio of forward/backward current densities) for Pt_3_Co/C is 1.62, higher than that for commercial Pt/C (1.15) and PtRu/C (1.06), indicating their high MOR durability^62, 63^, as supported by the chromoamperometric (CA) tests (SI Figure S4), which illustrate that the current density of Pt_3_Co/C for MOR keeps higher than that of commercial Pt/C and PtRu/C catalysts for the entire time course. The ECSA-based activities are usually used to measure the intrinsic catalytic behavior of Pt in different chemical environments. If we calibrate using the total mass of the catalysts loaded on the electrode, the anodic peak current density for Pt_3_Co/C is 1558.6 mA mg^−1^, also much higher than that for commercial Pt/C (461.3 mA mg^−1^) and PtRu/C catalysts (649.5 mA mg^−1^), as displayed by Fig. 4d. By optimizing the overall size of the Pt_3_Co alloy particles, we may further improve their catalytic performance for MOR. In addition, we may consider alloying Pt with other or multiple transition metals, e.g. Ni, Fe, and Cu, to enhance its electrocatalytic properties.

In summary, we reported our investigation on the synthesis of bimetallic PtCo alloy nanoparticles, their activation, as well as the catalytic performance for MOR. We emphasized the change in composition during the activation process. We initially prepared PtCo alloy nanoparticles with Pt/Co molar ratio of 1/2, and found that the activation by acetic refluxing could lead to significant loss of the Co component although the alloy nature could be maintained. The final PtCo alloy products have stable Pt/Co molar ratio of 3/1. The final carbon-supported Pt_3_Co alloy particles exhibit superior activity and stability for MOR, and their activity is 2.4 and 1.5 times of that for Pt/C (3.97 mA cm^−2^) and PtRu/C (5.53 mA cm^−2^), respectively.

## Methods

### General materials

Platinum (II) acetylacetonate (Pt(acac)_2_, 98%) and oleylamine (OLA, 95.4%) from J&K Scientific, cobalt(II) acetylacetonate (Co(acac)_2_, 97%), 1-octadecene (>90.0%), aqueous HClO_4_ solution (70%, ACS reagent) and Nafion 117 solution (5% in a mixture of lower aliphatic alcohols and water) from Aladdin Reagents, methanol (99%) and toluene (99.5%) from Beijing Chemical Works, Vulcan XC-72 carbon powders (XC-72C with BET surface area of ca. 250 m^2^ g^−1^ and average particle size of 40∼50 nm) from Cabot Corporation, and commercial Pt/C (20 wt% of Pt nanoparticles on Vulcan XC-72 carbon supports) and PtRu/C (supported on XC-72, 30 wt% in total mass and Pt:Ru = 2:1) from Johnson Matthey, were used as received.

### Synthesis of Co, Pt, and PtCo nanoparticles

We conduct the synthesis of Pt, Co, and PtCo alloy nanoparticles in a three-necked flask fitted with a condenser and a stir bar. In detail, for Co and Pt, we add 0.1 mmol (25.7 mg) of Co(acac)_2_ (or 0.05 mmol (20 mg) of Pt(acac)_2_, or 0.1 mmol of Co(acac)_2_ and 0.05 mmol of Pt(acac)_2_), 10 mL of 1-octadecene, and 2.5 mL of oleylamine to the reaction container. We then bring the temperature of this mixture to 200 °C and keep this temperature under flowing N_2_ for 2 h to reduce the Co or Pt ions by oleylamine. Subsequently, we precipitate the Co, Pt, or PtCo nanoparticles by methanol, collect them by centrifugation, purify them by washing with methanol, and re-disperse them in 20 mL of toluene.

### Loading of the PtCo nanoparticles on carbon supports

We load the PtCo alloy nanoparticles on carbon supports for the tests of their electrochemical behaviors toward MOR. We add a calculated amount of carbon powers to the PtCo colloidal solution in toluene, and stir the mixture for 24 h at room temperature. We subsequently collect the carbon-supported PtCo nanoparticles (labeled as PtCo/C) by centrifugation, wash them thrice with methanol, and dry them in vacuum at ambient temperature. The mass loading of Pt_3_Co on the carbon supports was determined to be 11.8% by inductively coupled plasma atomic emission spectrophotometry (ICP-AES, Perkin-Elmer Optima 5300DV spectrometer). Then we re-disperse the PtCo/C catalysts into 20 mL of acetic acid by ultra-sonication, and reflux the mixture for 3 h at 120 °C to remove the capping agents from the particle surface^43^. Finally, we recover the PtCo/C catalysts from acetic acid by centrifugation, wash them thrice with water, and dry them at room temperature in vacuum.

### Particle characterizations

We determine the size and morphology of the Co, Pt, and PtCo nanoparticles by a JEOL JEM-2100 transmission electron microscopy (TEM) operated at 200 kV. We use an energy dispersive X-ray spectroscopy (EDX) analyzer attached to the TEM operated in the STEM mode to analyze the compositions of the as-prepared nanoparticles. We record the powder X-ray diffraction (XRD) patterns on a Bruker D8 diffractometer using Cu Kα radiation (λ = 0.154056 nm), and obtain the X-ray photoelectron spectra (XPS) of the components in the nanoparticles using a Thermo Scientific K-Alpha XPS spectrometer.

### Electrochemical measurements

We carry out the electrochemical measurements on a standard three-electrode cell connected to a Bio-logic VMP3 (with EC-lab software version 9.56) potentiostat. We use a leak-free Ag/AgCl (saturated with KCl) electrode and a Pt mesh (1 × 1 cm^2^) as reference electrode and counter electrode, respectively. For the preparation of working electrode, we ultrasonically disperse 4 mg of PtCo/C in a mixture containing 1.98 mL of ethanol and 0.02 mL of Nafion solution. We then dispense 5 ul of the ink onto the 5 mm of glassy carbon disk electrode, and then dry the electrode in a stream of warm air at 70 °C for 1 h.

We determine the electrochemically active surface area (ECSA) of the catalysts by recording the room temperature cyclic voltammograms of PtCo/C in argon-purged HClO_4_ (0.1 M). The potential range is −0.25 V–1 V with sweeping rate of 50 mV s^−1^. We also use cyclic voltammetry with a potential range of 0–1 V and a scanning rate of 20 mV s^−1^ to evaluate the performance of PtCo/C catalysts in room-temperature MOR. The electrolyte is 1 M methanol in 0.1 M aqueous HClO_4_ solution.

## Electronic supplementary material

Supplementary Information
