# Supplementary material for: Uniformly dispersed platinum-cobalt alloy nanoparticles with stable compositions on carbon substrates for methanol oxidation reaction
Source: Sci Rep. 2017 Sep 12;7:11421. doi: 10.1038/s41598-017-10223-2 (PMC5595832; doi:10.1038/s41598-017-10223-2)
Supplement: Supplementary file 1 — Supplementary Information [file 41598_2017_10223_MOESM1_ESM.doc]

Supplementary Information

**Uniformly dispersed platinum-cobalt alloy nanoparticles with stable compositions on carbon substrates for methanol oxidation reaction**

Hui Liu^1,2^, Chengyin Li^1^, Dong Chen^1^, Penglei Cui^1^, Feng Ye^1^, and Jun Yang^1,2,3,*^

^1^State Key Laboratory of Multiphase Complex Systems, Institute of Process Engineering, Chinese Academy of Sciences, Beijing, China 100190. Tel: 86-10-8254 4915; Fax: 86-10-8254 4915; [jyang@ipe.ac.cn](mailto:jyang@mail.ipe.ac.cn)

^2^Center for Mesoscience, Institute of Process Engineering, Chinese Academy of Sciences, Beijing 100190, China

^3^University of Chinese Academy of Sciences, No. 19A Yuquan Road, Beijing, 100049, China

We gratefully acknowledge the financial supports from the National Natural Science Foundation of China (Nos.: 21376247, 21476246, 21506225, 21506234, 21573240) and Center for Mesoscience, Institute of Process Engineering, Chinese Academy of Sciences (COM2015A001).

**Figure S1.** XRD patterns of bimetallic PtCo nanoparticles before (a) and after (b) refluxing in acetic acid at 120°C as well as the Pt_3_Co reference with JCPDS Card No. of 290499 (c).

**Figure S2.** EDX analyses of bimetallic PtCo nanoparticles before (a) and after (b) refluxing in acetic acid at 120°C.

**Figure S3.** TEM images of the commercial Pt/C catalyst (a), which consists of 20 wt% of Pt nanoparticles (ca. 3.5 nm) on Vulcan XC-72 carbon supports, and commercial PtRu/C catalyst (b), which consists of 30 wt% of PtRu alloy particles (ca. 3 nm) on Vulcan XC-72 carbon supports.

**Figure S4.** Chronoamperograms of the Pt_3_Co/C, commercial Pt/C, and commercial PtRu/C catalysts at 0.45 V vs Ag/AgCl in argon-purged HClO_4_ (0.1 M) with 1 M methanol.
